# Spatio-temporal variability in forest biodiversity associated with human well-being across socio-economic deprivation gradients

**DOI:** 10.1038/s41559-025-02765-w

**Published:** 2025-06-24

**Authors:** J. C. Fisher, M. Dallimer, G. E. Austen, K. N. Irvine, S. G. Aizlewood, P. M. King, H. A. Jackson, R. D. Fish, Z. G. Davies

**Affiliations:** 1 Durrell Institute of Conservation and Ecology (DICE), School of Anthropology and Conservation, University of Kent, Canterbury, UK; 2 Sustainability Research Institute, School of Earth and Environment, University of Leeds, Leeds, UK; 3 Centre for Environmental Policy, Imperial College London, London, UK; 4 Social, Economic and Geographical Sciences Department, James Hutton Institute, Aberdeen, UK; 5 Woodland Trust, Kempton Way, Grantham, UK; 6 UK Centre for Ecology and Hydrology, Lancaster Environment Centre, Bailrigg, UK

**Keywords:** Environmental studies, Ecosystem services, Psychology

## Abstract

Biodiversity declines are accelerating globally, impacting ecosystem functioning, with consequences for human health. Interactions with biodiversity can be associated with human well-being benefits at the individual level, leading to substantial gains for society when scaled up across populations. However, existing research has not accounted for the species within ecological communities and their effect traits (for example, colours, sounds) that can elicit well-being responses. Many species’ effect traits are seasonal, and spatial variation in exposure to ecosystems by different sectors of society can lead to unequal opportunities to gain well-being. Here we use an interdisciplinary analytical approach to explore how the association between forest biodiversity and well-being fluctuates: (1) temporally, between different seasons and (2) spatially, across socio-economic deprivation gradients at a national scale (England and Wales). Species’ effect traits and participant well-being were derived through a series of seasonal participatory workshops and questionnaires that incorporated BIO-WELL (a biodiversity–well-being psychometric scale). By generating spatially explicit data, we could examine variability in forest biodiversity associated with human well-being across socio-economic deprivation gradients. Forest species’ effect trait richness was spatially heterogeneous, particularly in autumn, spring and summer. Broadleaf forests had greater species’ effect trait richness than other categories of forest. Forests with higher species’ effect trait richness and forests that were associated with higher self-reported participant well-being were in areas with the least socio-economic deprivation. Forest creation/restoration and nature–health interventions must recognize this ecological and social diversity to ensure initiatives are equitable and socially just.

## Abstract (web summary)

Applying a combined social science and trait-based ecology approach, the authors identify ecological traits in forests eliciting positive or negative well-being among human participants in England and Wales and find that forests with higher species’ effect trait richness, and those associated with higher participant well-being, are in areas with the least socio-economic deprivation.

## Main

Biodiversity declines are accelerating globally^1^. This loss of biodiversity is impacting the stability and functioning of ecosystems, with potentially far-reaching consequences for human health and well-being^2^. Well-being is a multidimensional concept, encompassing different contributions to human quality of life^3^. The World Health Organization conceptualizes well-being as “a state of complete, physical, mental and social well-being and not merely the absence of disease or infirmity”^4^. The multiple domains of well-being encompassed in this definition (bio-, which is physical; psycho-, which is mental, consisting of both cognitive and emotional; and social) comprise the ‘biopsychosocial’ model of health that originates from integrative medicine^5^. An expanded version of this model, called the biopsychosocial–spiritual model^6,7^, also includes a spiritual domain, conceived as including a connection to something greater than oneself.

Spending time in ecosystems such as forests and wetlands has been linked to a multitude of benefits such as reduced stress, improved cognition and better quality of life^8–10^. Given that well-being predicts mortality and morbidity^11^, scaling up these individual-level gains across entire populations could support the public health sector through substantial avoided societal and healthcare costs (for example, refs. ^12,13^). Therefore, improving our understanding of how exposure to biodiversity can promote well-being is likely to have widespread implications for both public health and conservation, via initiatives such as nature-based solutions and social (‘green’) prescribing interventions^14,15^.

Whereas an extensive literature has established that interactions with nature can generate positive well-being responses, this existing body of research generally takes a simplistic approach that relies on homogeneous measures of exposure to ‘greenspace’ or ‘greenness’^9,16^. The role biodiversity plays in delivering improved health has been largely overlooked^17^. This is despite people’s engagement with biodiversity within ecosystems being multisensory^18^ and influenced by personal and cultural associations^19,20^. Without accounting for biodiversity, and how it is experienced and/or perceived, we may not be able to conserve, restore or create ecosystems that will also generate greater benefits for human health and well-being.

These complex biodiversity–human health relationships can be examined through a functional ecology lens. Some species traits, known as ‘effect traits’, underpin ecosystem service delivery^21^. For example, mean diameter at breast height of a tree is linked to carbon storage^22^. Likewise, the species’ traits that lead to changes in people’s well-being can be considered effect traits (for example, the ‘calling’ sounds of tawny owls (*Strix aluco*) and ‘prickly’ texture of brambles (*Rubus fruticosus*) eliciting positive and negative well-being, respectively)^17^. Different ecosystems will thus provide different levels of well-being, based on the array of species that occur within the ecological community and the effect traits they support.

Ecosystem impacts on health and well-being fluctuate over time^23^. For instance, grass pollen causes allergies leading to asthma and rhinitis (hay fever), which can be tracked over the course of the year and spatially^24^. Similarly, bird communities alter intra-annually, influencing the supply of cultural ecosystem service benefits^25^. This reflects the seasonality of biodiversity in any given ecosystem, where variations in temperature and precipitation affect resource availability and, subsequently, the presence, abundance and diversity of species and the effect traits they support. Seasonal phenological events themselves, such as leaf senescence in deciduous trees, have also been shown to stimulate positive emotions^26^. Moreover, seasonality also influences how people use ecosystems (for example, ref. ^27^), due to weather or cultural activities such as participation in holidays and festivals. Despite this, temporal variability is rarely considered in nature–human health research^23^.

Spatial variation in exposure to ecosystems by different sectors of society can lead to unequal opportunities to gain well-being (often referred to as ‘environmental health inequalities’). In Europe, for instance, socio-economically deprived groups are often less exposed to green/blue spaces and have a higher prevalence of poor health outcomes^28^. Consequently, they could benefit disproportionately from access to such ecosystems. For example, Mitchell et al.^29^, demonstrated that access to recreational greenspace was positively associated with improved mental well-being across the United Kingdom and more so for those under greater financial strain. However, the evidence base is inconclusive and contradictory (Schüle et al.^30^ provides a review). Having a deeper insight into the distribution of species’ effect traits within the ecosystems people visit could help disentangle these equivocal findings.

**Fig. 1:**
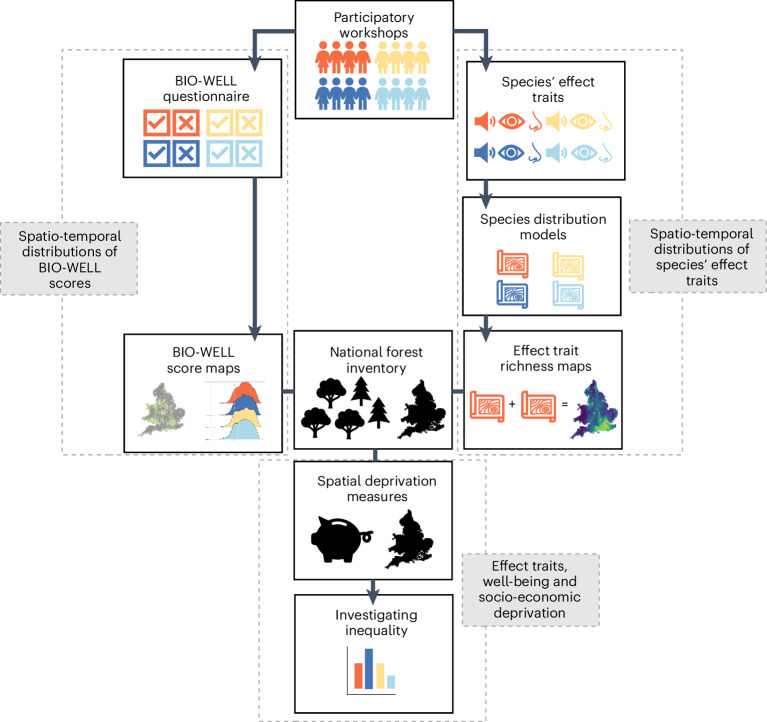
Diagram of our methodological steps. Grey boxes and dashed lines show the different study aims. White boxes indicate the sequential stages of data collection and/or analysis. Colours represent data collection and/or analyses that were seasonal (orange = autumn, dark blue = winter, yellow = spring, light blue = summer).

Here we use a novel analytical approach to explore how associations between biodiversity and well-being fluctuate: (1) temporally, between different seasons and (2) spatially, at a national scale (England and Wales) and across socio-economic gradients (Fig. 1). Specifically, we focus on forest ecosystems, which have declined in global land area by over 30% between 1990 and 2015^31^, yet support 80% of terrestrial biodiversity^32^. Temperate forests cover 16% of global land area and are less intact in regions with high human population density and intensive agriculture^33^. Consequently, they are commonly the focus of restoration and creation initiatives, often with the aim of producing ‘triple wins’ for climate change mitigation, biodiversity and human well-being^34,35^.

**Extended Data Fig. 1:**
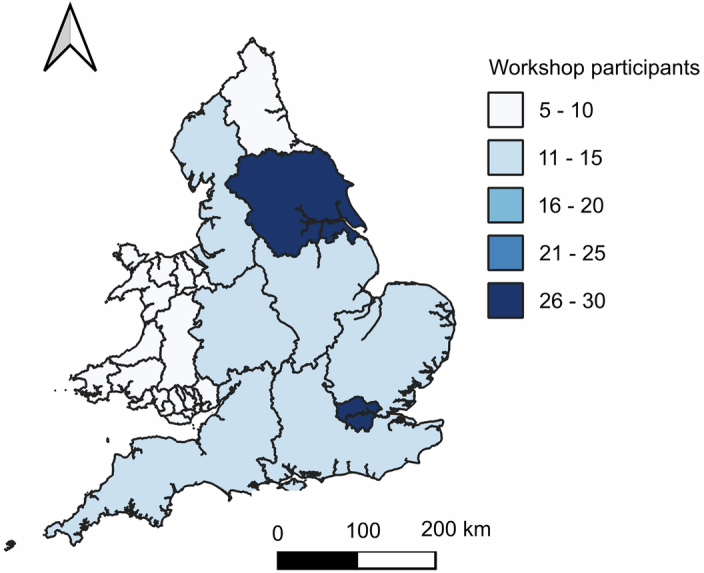
Residential location of workshop participants across England and Wales. Blue shading represents the number of participants sampled from each region. Base map adapted from GADM.

**Extended Data Fig. 2:**
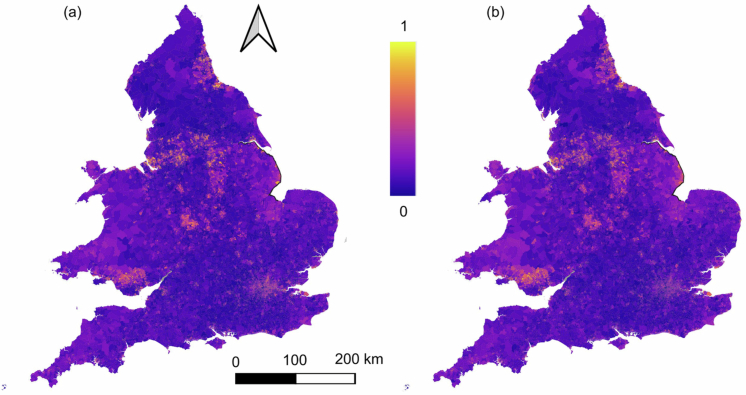
Spatial distribution of income- and employment-related deprivation in England and Wales. The gradient shows the proportion of the public considered to be experiencing (**a**) income- or (**b**) employment-related deprivation living in an area. Base maps adapted from GADM.

We conducted a large, participatory process with a diverse cross-section of the public from England and Wales (Extended Data Fig. 1) in each of the four seasons (autumn, winter, spring and summer). This enabled us to identify which forest species, and their effect traits (colours, sounds, smells, textures and behaviours), were described by participants in relation to both positive and negative well-being at different times of the year^17^. We examined the five domains that constitute the biopsychosocial–spiritual model of health^5,6^: (1) physical (the body and how someone feels physically); (2) emotional (positive and negative mood); (3) cognitive (state of mind); (4) social (perceived connections with others) and (5) spiritual (relationships with something greater than oneself). Hereafter we use the term ‘well-being’ in relation to people’s biopsychosocial–spiritual responses to forest biodiversity. Using species distribution models (SDMs), we created spatio-temporal distributions of species’ effect traits. Additionally, we quantified the spatially explicit self-reported well-being responses people derive from forest biodiversity, using a questionnaire that incorporated the biodiversity–well-being psychometric scale BIO-WELL^18^ (https://research.kent.ac.uk/bio-well/). We therefore examined associations between biodiversity and human well-being spatio-temporally in two ways: through the species’ effect traits and via BIO-WELL (Fig. 1). To investigate environmental health inequalities, we used government data on income- and employment-related deprivation, mapped at the finest spatial resolution that is publicly available (Extended Data Fig. 2). We then coupled the distributions of species’ effect traits and BIO-WELL scores with socio-economic deprivation.

## Results

### Seasonal species’ effect traits

**Fig. 2:**
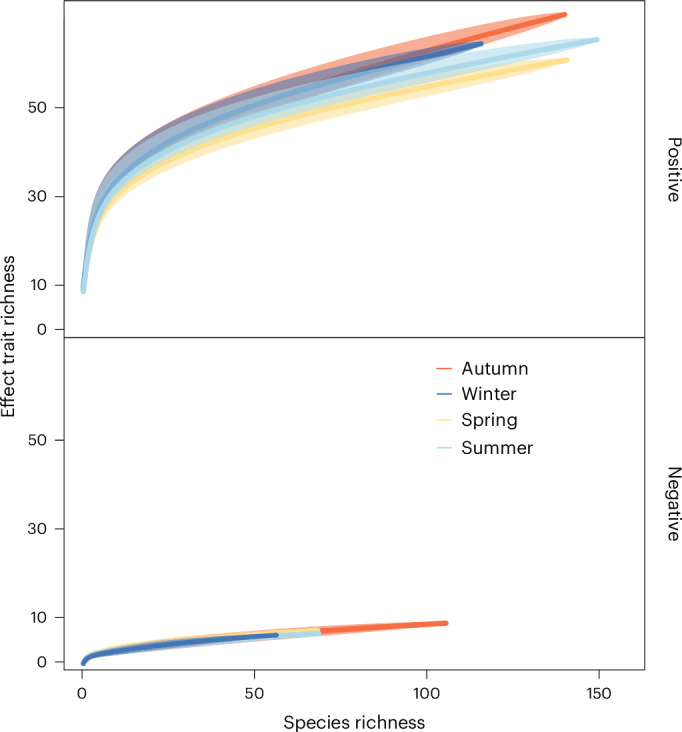
Accumulative curves between species richness and species’ effect trait richness associated with positive and negative well-being per season in forests across England and Wales. The curve displays the mean, with the upper and lower bounds of the shaded area representing 95% confidence intervals (mean value ± standard error).

We identified 78 species’ effect traits that were described by participants as eliciting some form of positive or negative well-being across autumn, winter, spring and summer (Fisher et al.^17^ includes the full list of all species’ effect traits), associated with the 131 forest species that we could generate SDMs for (that is, they had sufficient fine-scale resolution spatial presence/absence data available and/or model fit was acceptable). Most of these species’ effect traits (69) were linked with positive rather than negative (9) well-being, and four were allied to both. The richness of effect traits increased with the number of species, particularly in autumn and winter (Fig. 2).

### Spatio-temporal distributions of species’ effect traits

**Fig. 3:**
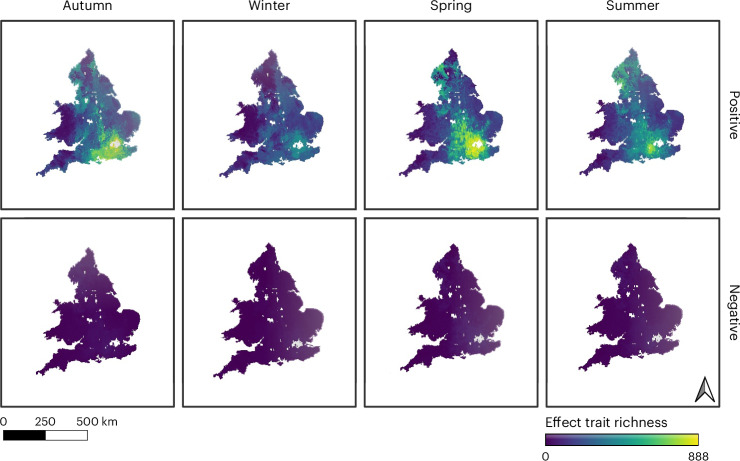
Spatio-temporal distributions of cumulative forest species’ effect trait richness per pixel across England and Wales for positive and negative well-being. Cumulative species’ effect trait richness is the total number of unique effect trait–well-being incidences across all species, separated into positive and negative well-being, in autumn, winter, spring and summer. Base maps adapted from GADM.

We combined the SDMs with the number of species’ effect traits per species that were described in relation to positive or negative well-being responses, to create eight spatio-temporal maps, one per season (Fig. 3). These maps showed high levels of spatial heterogeneity, with cumulative species’ effect trait richness (the total number of unique effect trait–well-being incidences across all species) ranging from zero to 888 for positive well-being and from zero to 66 for negative well-being.

**Fig. 4:**
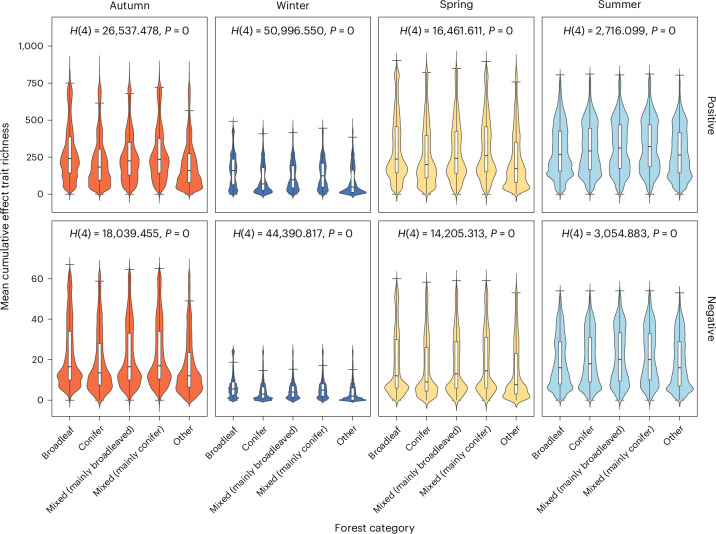
Mean cumulative species’ effect trait richness per pixel for different categories of forest across England and Wales. Forest categories (*n* = 566,394 forest polygons) follow NFI definitions; Supplementary Table 1. Violin plots display the probability density of mean cumulative species’ effect trait richness (the width representing the frequency of data points) for each forest category, associated positive or negative well-being per season. White boxplots within the violin plots show the median, interquartile range, minimum and maximum of the same data. Kruskal Wallis H statistics are given in each panel, used to test for differences between forest categories per season, for positive and negative well-being, respectively (Supplementary Table 2 provides post-hoc Dunn–Bonferroni test results). Note: the *y* axis for negative well-being is a smaller scale.

**Extended Data Fig. 3:**
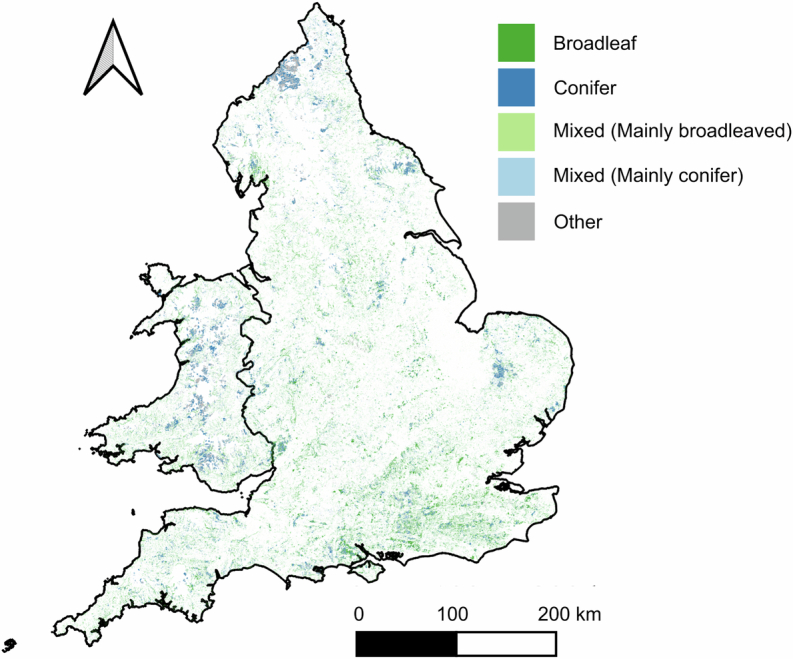
Distribution of National Forest Inventory forest categories across England and Wales. Colours represent forest categories (green = broadleaf, 60%, blue = conifer, 14%, light green = mixed mainly broadleaved, 5%, light blue = mixed mainly conifer, 5%, dark grey = other, 17%). Base map adapted from GADM.

Hotspots of species’ effect traits that elicit positive well-being were apparent across southeast England, broadly coinciding with where broadleaf forest is predominately located (Extended Data Fig. 3). Indeed, we found significantly different cumulative species’ effect trait richness between all forest categories (following National Forest Inventory (NFI) definitions; Supplementary Tables 1 and 2). In summer, forests of all categories contained a significantly higher mean cumulative species’ effect trait richness than for the other three seasons but most notably compared to winter. Broadleaf forests had significantly greater mean cumulative richness of species’ effect traits that were associated with positive well-being compared to other forest categories in autumn, winter and spring (Fig. 4 and Supplementary Table 2). In summer, coniferous forests had the highest mean cumulative richness of species’ effect traits. ‘Other’ forest categories had the lowest mean cumulative richness of species’ effect traits in every season. The patterns for mean cumulative species’ effect traits associated with negative well-being were consistent with those found for positive.

### Spatio-temporal distributions of BIO-WELL scores

**Fig. 5:**
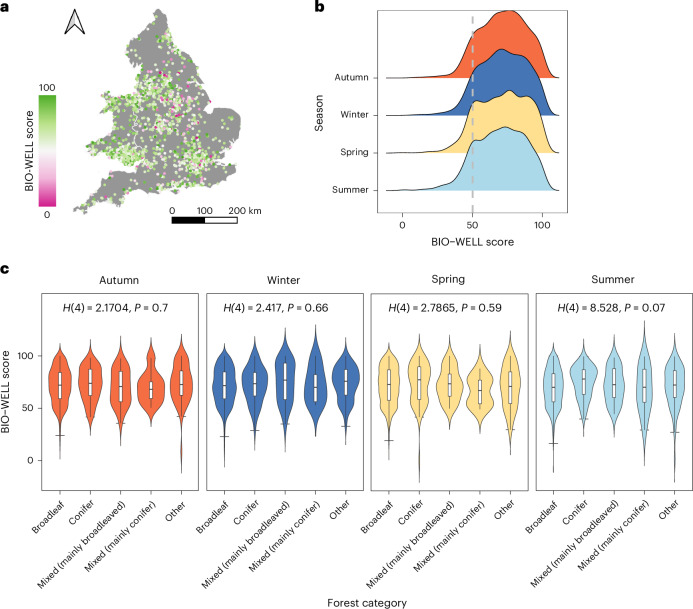
BIO-WELL scores, indicating well-being responses to forest biodiversity for online questionnaire participants across England and Wales. **a**,**b**, Spatial distribution of BIO-WELL scores coloured by value (**a**) and a smooth histogram of BIO-WELL scores for each season (**b**). Dashed grey line represents the midpoint above or below which biodiversity is associated with positive (>50) or negative (<50) well-being responses, respectively. **c**, Violin plots displaying the probability density of BIO-WELL scores (the width representing the frequency of data points) for each forest category. Participants indicated the location of the nearby forest that their BIO-WELL score was relevant to in the questionnaire (*n* = 4,197). White boxplots within the violin plots show the median, interquartile range, minimum and maximum of the same data. BIO-WELL scores >50 and <50 indicate positive and negative well-being responses, respectively. Kruskal Wallis H statistics are given in each panel, used to test for differences between forest categories per season (Supplementary Table 4 provides post-hoc Dunn–Bonferroni test results). Base map in **a** adapted from GADM.

In total, 4,197 participants fully completed our online questionnaire, with different participants per season. Each seasonal cohort represented a diverse socio-demographic of the English and Welsh public in terms of gender, age, ethnicity and education (Supplementary Table 3), being distributed across England and Wales in both rural and urban areas (Fig. 5). Overall, participants experienced positive well-being in response to forest biodiversity, with BIO-WELL scores averaging 71.1 (range: 0.2–100 out of a possible 0–100), where values <50 indicate a negative response to biodiversity (12% of participants) and >50 indicate a positive response (88% of participants).

In general, we did not detect any differences in the BIO-WELL scores of participants between forest categories or across seasons (Fig. 5c and Supplementary Table 4). The one exception to this was summer, where participants reported statistically higher BIO-WELL scores associated with coniferous forest.

### Effect traits, well-being and socio-economic deprivation

**Fig. 6:**
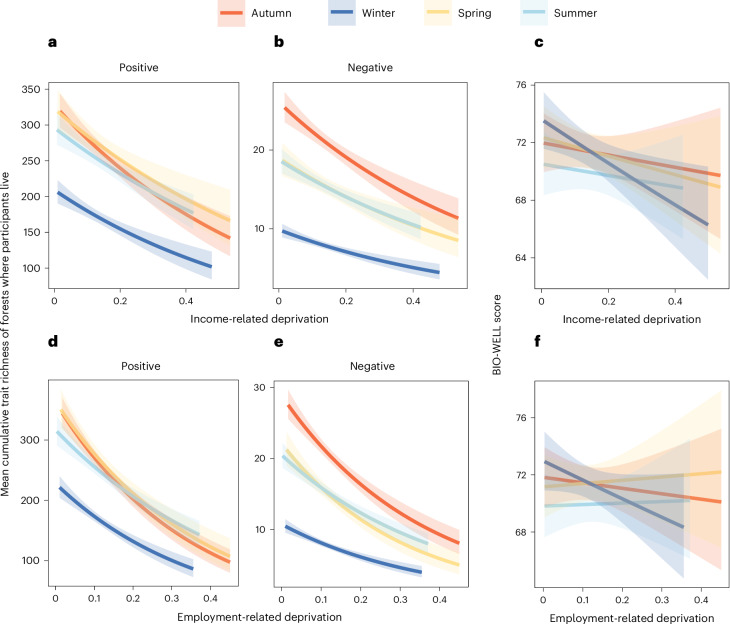
Associations between mean cumulative species’ effect trait richness and participant BIO-WELL scores per season across socio-economic deprivation gradients in England and Wales. **a**–**f**, The *y* axes represent either the mean cumulative species’ effect trait richness of all forests within each area where participants live in England and Wales, eliciting positive (**a**,**d**) or negative (**b**,**e**) well-being or BIO-WELL scores (**c**,**f**) (where >50 denotes positive well-being responses to forest biodiversity and <50 is negative). The *x* axes are the proportion of the public considered to be experiencing income- or employment-related deprivation living in an area. Forest area (ha) is included as a covariate. Slopes indicate a general linear model with a 95% confidence interval (coloured shading) (Supplementary Table 5). Note: the *y* axes for negative well-being (**b**,**e**) are a smaller scale than for positive (**a**,**d**) and the *y* axis for BIO-WELL (**c**,**f**) is restricted.

**Extended Data Fig. 4:**
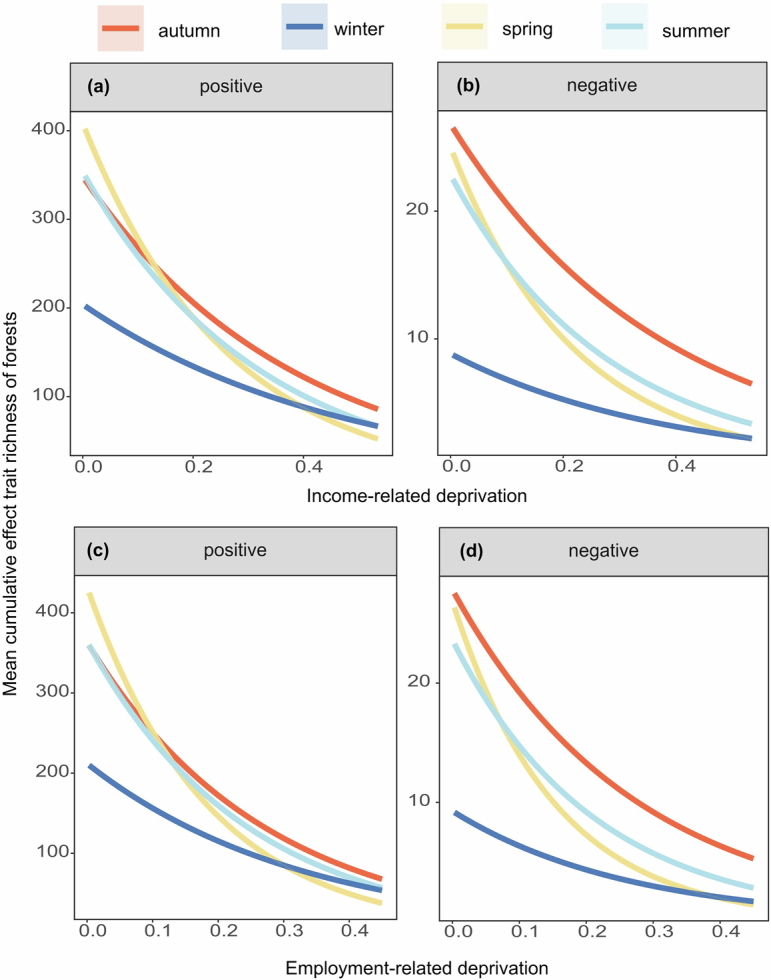
Associations between mean cumulative species’ effect trait richness, per season, across socioeconomic deprivation gradients in England and Wales. The y axes represent the mean cumulative species’ effect trait richness of all forests across England and Wales, eliciting positive (**a, c**) or negative (**b, d**) wellbeing. The x axes are the proportion of the public considered to be experiencing income- or employment-related deprivation living in an area. Forest area (hectares) is included as a covariate. Slopes indicate a general linear model with a 95% confidence interval (coloured shading) (Supplementary Table 6). NB: the y axis for negative wellbeing (**b**, **d**) is a smaller scale.

There was no association between forest area and level of deprivation (β = 0.027, 95% CI = −0.996–1.051). However, mean cumulative species’ effect trait richness, for both positive and negative well-being and across all four seasons, was greatest in the least socio-economic deprived areas where participants lived (Fig. 6a–d and Supplementary Table 5a). These patterns were consistent when examined for all forests across England and Wales (Extended Data Fig. 4 and Supplementary Table 6). Participant BIO-WELL scores were negatively associated with income-related deprivation in the winter and spring (Fig. 6e,f and Supplementary Table 5b).

## Discussion

To understand how to create and manage ecosystems to meaningfully improve human health and well-being associated with biodiversity, researchers must move beyond coarse measures of ‘nature’ and ‘greenspace’ and account for the ecological communities present, which are inherently dynamic and spatially variable. In addition to better representing the complexity of ecosystems, we also need to recognize that human populations are diverse in their socio-economic composition and not distributed in a homogeneous manner. In this paper, we therefore begin the process of teasing apart this intricacy by examining spatio-temporal patterns between mean cumulative forest species’ effect trait richness, well-being (both positive and negative) associations with biodiversity and socio-economic deprivation gradients. Where we cannot infer causality from our statistical analyses, our approach demonstrates how evidence derived from participatory processes and quantitative social science research methods^17,18^ can initiate a step change in the development of forest restoration initiatives that seek to benefit both people and biodiversity.

We used two complementary ways to examine the potential for human well-being associated with forest biodiversity: species’ effect traits and BIO-WELL. The former takes a functional ecology perspective^17,36^ and the latter an environmental psychological standpoint^18^. We show that there are higher numbers of species’ effect traits where species richness is greater, far more so for positive compared with negative well-being, across all seasons. Moreover, this pattern is more pronounced for autumn and winter for positive well-being, with the implication being that improvements in forest biodiversity would have a relatively larger impact on enhancing positive species’ effect traits in these seasons (for example, refs. ^37,38^). When spatio-temporal distributions of mean cumulative species’ effect traits were examined, summer supported the greatest richness of traits associated with positive well-being responses. Regional variation was observed, with the southeast having higher densities of traits. This reflects wider patterns of biodiversity across Britain, where the majority of species are on the northwest edge of their geographic range^39^ and where broadleaf forest and ancient woodlands are concentrated^40^. With relatively few negative species’ effect traits identified, little spatio-temporal variation was detected. Our findings affirm wider evidence suggesting that diverse forest ecosystems can help to positively enhance people’s well-being^8,9^. This is despite the data requirements of the SDMs inevitably limiting the number of species that could be mapped to those that are part of national recording schemes with standardized survey methods, with adequate numbers of presence/absence records and that produced statistically acceptable models^41,42^. Furthermore, SDMs are not an actual representation of the biodiversity that is present in specific forests. Given that only 7% of Britain’s forests are in good ecological condition (for example, presence of deadwood, veteran trees and diversity of ecological niches that support biodiversity^40,43^), it is unlikely that the majority are currently delivering their full human well-being potential. This adds further weight to calls for conservation and nature recovery to be at the heart of forest restoration initiatives^44,45^.

Further research is needed to ascertain the degree to which particular species’ effect traits are more or less beneficial than others for well-being (for example, the smell of coniferous trees compared to the rough texture of bark), whether the relative ‘strength’ of each particular effect trait leads to different levels of well-being (for example, light to dark purple, potentially equating to within-species phenotypic variation) or if/how multiple effect traits interact to result in additive or multiplicative well-being responses (the cumulative effect of watching adult birds provisioning their chicks alongside the sound of birdsong from one or more species). Understanding these details could facilitate more targeted public health recommendations and interventions (for example, ref. ^46^) and ally research in this field with that with investigating how different levels of, and interactions between, multiple effect traits influence regulating and provisioning ecosystem service benefits^21,22^.

We found evidence of environmental health inequalities, with the more deprived sectors of society in England and Wales having less potential to gain positive well-being associated with forest biodiversity in proximity to where they live. For instance, disparities were apparent within southeast England between inland and coastal areas, the latter being typically suffering from more extreme levels of socio-economic deprivation^47^. Such spatial inequalities may be further exacerbated by the fact that the green spaces, where they do exist, are either not accessible to the public or are visited infrequently^48^. The lower use of green spaces can be attributed to a variety of factors that are social (for example, personal safety concerns), individual (for example, confidence in managing children outdoors) and contextual (for example, no free time)^49^. When we examined patterns in seasonality, people living in more deprived areas reported lower BIO-WELL scores in winter and spring. This trend could reflect the reduced species’ effect trait richness apparent in winter but also less engagement with forests during the colder seasons of the year (for example, poor weather^50^).

The unequal distribution of forests rich in mean cumulative species’ effect traits associated with positive well-being across deprivation gradients could also be explained by the ‘luxury effect’, predominately characterized as an urban phenomenon, which describes a positive association between higher biodiversity and affluence^51^. It is characterized by wealthier residents being drawn to more biodiverse and/or greener areas, creating a demand that raises property values and rents that effectively ‘price out’ individuals on lower incomes^51^. Another possible hypothesis could be that local authorities and/or private property owners in deprived areas invest less in forest conservation^52^. At local scales, however, more nuanced spatial patterns could be apparent that would require a finer-resolution analysis to disaggregate (for example, community forests in deprived areas may have relatively high biodiversity).

In areas of England and Wales characterized by higher deprivation, access to forests could be improved by strategically targeting nature recovery through better management of existing ecosystems and the creation of new ones. This is particularly pertinent, given that current restoration and tree-planting regimes that are intended to augment carbon sequestration generally overlook the heterogeneous biodiversity preferences, perspectives and values of people who may interact with the forests^32,34,53,54^. The success of forest creation/restoration projects relies on recognizing this diversity, ensuring that such initiatives are equitable and socially just. In turn, this means that they are more likely to be supported by, and benefit, the local community^55,56^. Nevertheless, making management decisions within forests to promote species’ effect traits that have the potential to enhance human well-being needs to be balanced alongside ecological considerations. For example, removing the species and effect traits associated with negative well-being could have detrimental consequences for ecosystem functioning and ecosystem service delivery (for example, degrading trophic interactions). Likewise, culturally important or charismatic species may not be of conservation interest or could be non-native^57^. Trade-offs may need to be navigated, taking care to ensure that unintended adverse impacts for biodiversity conservation are avoided.

In all seasons, we found that broadleaf forests had a greater richness of mean cumulative species’ effect traits compared with other forest categories. In temperate climates, people visiting deciduous forests in autumn are more likely to encounter fruiting fungi or senescence of trees (for example, ref. ^58^), whereas those visiting in spring may experience forest-floor flowers^40^. Deciduous trees themselves support disproportionately high numbers of species’ effect traits that elicit positive well-being responses^17^. This is because effect traits linked to the phenology and longevity of deciduous trees are embedded in people’s everyday lives, for instance, large, old charismatic trees in Finland promoted sensory and emotional experiences^59^. Deciduous old-growth trees provide habitat for the highest diversity of animals, plants and fungi compared to other forest types^40,60^. Nonetheless, increasing the biodiversity of all forests has the potential to enhance human well-being and possibly delivering additional benefits across multiple other classes of ecosystem service^22^. BIO-WELL scores did not vary significantly between forest categories or seasons, other than being higher for coniferous forests in summer^19^. This might be a consequence of the uneven distribution of forest categories (81% broadleaf, 8% conifer, 3% mixed broadleaf, 1% mixed conifer, 7% other) in the analysis. On the other hand, coniferous forests in summer also supported the highest richness of species’ effect traits. For example, at this time of year, coniferous forests tend to be characterized by a pine scent that often carries cultural and personal importance and a unique canopy structure that can create well-defined and sun-lit footpaths^61,62^.

In this Article, we make new advances in biodiversity–well-being research by drawing upon and integrating functional ecology and environmental psychology concepts. Foremost, our granular approach can be operationalized by those planning where, when and for whom forest protection, restoration and creation should be targeted^63^. Likewise, it can inform the design and practice of social ‘green’ prescribing interventions^14,15^, particularly with the goal of improving well-being from forest biodiversity for certain sectors of society and to ensure that this is done adequately across the seasons. Indeed, moving forwards, a focus on how to improve people’s well-being from biodiversity in the colder seasons could prove especially fruitful. Practitioners delivering forest restoration/creation or social prescribing interventions could then use BIO-WELL to monitor changes in human well-being in response to the ecological condition of forests^18^. Replicating our methodology for non-forest ecosystems will provide a more comprehensive picture of the well-being potential of biodiversity at a landscape scale. Areas with poor forest species’ effect trait richness may contain other highly valuable ecosystems (for example, coastal grasslands) that support different ecological communities with their own suite of effect traits. Understanding and accounting for this complexity could create more opportunities to deliver natural environments that underpin healthier individuals and societies.

## Methods

### Study system

Britain’s forest ecosystems, which are 49% broadleaf and 51% coniferous, provide critical habitat for biodiversity^40^. Across England and Wales, 24.9% of the total land area is forest^43^. Forests are often publicly accessible and are among the most frequently visited types of ecosystem^48^. Here we use the NFI definition of forests (at least 0.5 ha in area, 20 m width and at least 20% canopy cover), using an open access dataset from the UK Government’s Forest Research department (www.forestresearch.gov.uk) (Supplementary Table 1 and Extended Data Fig. 3).

### Participatory workshops

We held four participatory workshops in 2019, to identify how people (*n* = 194) relate to forest biodiversity for their well-being^17,18,20,53^, during each of the four seasons (autumn *n* = 48, winter *n* = 50, spring *n* = 46 and summer *n* = 50). The participant cohort was new for each workshop. They represented a diversity of the public across age (18–29 years old *n* = 60, 30–59 *n* = 68, 60+ *n* = 66), ethnicity (white British *n* = 146, other *n* = 48), gender (female *n* = 102, male *n* = 92), social grade (ABC1 *n* = 114, CDE2 *n* = 80) and urban–rural resident (urban *n* = 153, rural *n* = 41) (Extended Data Fig. 1). Social grade is defined as: AB (higher and intermediate managerial, administrative, professional occupations), C1 (supervisory, clerical and junior managerial, administrative and professional occupations), C2 (skilled manual occupations) and DE (semi-skilled and unskilled manual occupations, unemployed). All participants had to have been living in Britain for at least five years, irrespective of their nationality and were over 18 years old. Financial incentives (£100 per person per weekend) and upfront payment of expenses were used to support inclusive participation. Participants were recruited by a social research company to minimize the potential for self-selection bias (that is, individuals with a keen interest in nature).

We took participants to two forests (one being a mixed-deciduous and coniferous plantation, the other an ancient woodland), geographically located in the centre of Britain^17,18,20^. The forests were chosen to ensure that their objective physical and biological characteristics were diverse, both within and across the two ecosystems. We also made sure that the participants were not ‘local’ to either forest to minimize the impact that prior experience may have had on the their well-being responses to the objective biodiversity features of the sites. We ran a series of data collection activities designed to prompt discussion about forest biodiversity and what traits participants noticed (for example, smells, colours, textures, sounds, behaviours). These included an in situ scavenger hunt, ex situ focus groups and a series of ex situ image-based Q-methodology exercises (refs. ^17,18,20^ provide details). Activities were audio recorded and transcribed. Ethical approval was provided by the School of Anthropology and Conservation Research Ethics Committee, University of Kent (Ref: 009-ST-19). All participants provided informed consent before taking part in the research.

### Seasonal species’ effect traits

Workshop transcripts were analysed using NVivo (Version 12, QSR International Ply Ltd). We coded specific traits and how people’s well-being responded to these traits, both positively and negatively, using the five domains of the biopsychosocial–spiritual model of health^18^ (physical, emotional, cognitive, social, spiritual). For each trait, we then identified the species to which the participant was referring (for example, in the Q-methodology image or named by the participant) (ref. ^17^ provides details). Species that do not occur in British forests were excluded from the dataset. We made inferences about the species in cases were the participants mentioned specific phenological elements (for example ‘acorns’ were listed as English oak, *Quercus robur*). When participants alluded to traits associated with a taxonomic group of organisms (for example ‘spots’ on birds), we consulted reputable sources (Supplementary Table 7) to derive a list of species with that trait, excluding those that were too generic (for example, ‘green’ on plants). We then recorded the seasonal occurrence of species and their effect traits (for example, pied flycatchers, *Ficedula hypoleuca*, are not present in Britain in winter).

All data processing and statistical analyses were conducted in R (Version 4.2.0 (ref. ^64^)). To explore the relationship between species and effect traits in each season, we plotted accumulation curves of trait and species richness (function ‘accumcomp’ in package BiodiversityR^65^), for positive and negative well-being separately. Across species, there may be overlap in effect traits, meaning that there can be redundancy (where species delivering the same functions as others become functionally redundant/exchangeable) and complementarity (optimal combinations of species that deliver the maximum services) within ecological communities^17^.

### Spatio-temporal distributions of species’ effect traits

We gathered 2019 seasonal occurrence records for England and Wales for species within taxonomic groups (birds, butterflies, fungi, mammals and plants, including trees) that have national recording schemes with standardized survey methods (Supplementary Table 7). We used the UK Meteorological Office (www.metoffice.gov.uk/learning/seasons) definition of each season, which is based on the annual temperature cycle: autumn (1 September–31 November), winter (1 December–28/29 February), spring (1 March–30 May) and summer (1 June–31 August).

To generate the SDMs for each individual species, we selected a suite of biologically meaningful predictor variables (Supplementary Table 8), including elevation, precipitation and temperature data at 0.5 km resolution (30 arcseconds). These data are freely available from the BioClim dataset^66^ (function ‘getData’, package Raster^67^). Elevation data were converted into topographic ruggedness (function ‘tri’, package spatialEco^68^). Topsoil data for land cover, dominant grain size and mean soil nitrogen concentration were acquired from the Countryside Survey^69^. Topsoil data were resampled using bilinear interpolation for continuous data to match the resolution of precipitation and temperature data. For each species, we tested the full set of environmental predictors for collinearity using a step-wise procedure, where highly correlated variables (VIF > 3) were removed.

We approximated seasonal distributions of individual species across English and Welsh forests using ensemble modelling, following best-practice techniques^41,70–72^. Given that recommendations for a minimum number of records for SDMs varies depending on whether species are common/rare and generalist/specialist^41^, we only retained species with a minimum of 80 survey records (for examples, ref. ^41^), leaving a total of 131 species (Supplementary Table 7). Survey records were uploaded using functions that minimize spatial autocorrelation while maximizing data availability (‘load_occ’ function in the ‘SSDM’ package^72^). SDMs are sensitive to the type of algorithm applied to the data, so we fitted a suite of algorithms to derive statistical consensus among projections: classification tree analysis, generalized linear model and multivariate adaptive regression splines (‘ensemble_modelling’ function in the ‘SSDM’ package^72^). We generated presence-only models for each species from the occurrence records and pseudo-absences (randomly selected artificial data about where each species cannot be found), using default parameters in the SSDM package^72^. Whereas SDMs can overestimate the distribution of planted species, they are routinely used to predict where non-native species may occur^73^. To boost predictive power while maintaining computational efficiency, we ran ten replicates per model algorithm per species^74^ and required that models performed above a threshold value of >0.7 for area under the curve^75^. Model accuracy statistics were produced for all models and evaluated using the true skill statistic, assessing values > 0.4 as fair, >0.5 as good, >0.7 as very good, >0.85 as excellent and >0.9 as perfect^76^. We created binary presence–absence maps using the highest true skill statistic threshold available for each species’ set of models. Binary maps were subsequently clipped to the NFI shapefile for forests in England and Wales.

Our species’ effect trait richness spatio-temporal distributions were constructed via the binary species maps (that is, the colour red was considered to be present wherever a European robin, *Erithacus rubecula*, was present). Traits were not treated as substitutable, given that the well-being benefits derived from traits are linked to the species and taxonomic group that the trait is supported by^17^. The spatio-temporal distributions of species’ effect trait richness were captured in eight maps (positive and negative well-being separately for each of the four seasons) for which the data represents the overall cumulative effect trait richness within each pixel (0.5 min of a degree). We extracted the mean cumulative species’ effect trait richness values across each NFI forest category using the ‘extract’ function in the package Raster^67^. We compared mean cumulative species’ effect trait richness between forest categories using a Kruskal Wallis H test and post-hoc Dunn–Bonferroni tests.

### Online seasonal questionnaire

In 2021, we administered an online questionnaire to 4,710 participants across England and Wales using Qualtrics, across the four seasons. Participants could only complete the questionnaire once across all four seasons and were not sent the questionnaire if they had been present at one of the workshops. Once again, participants were recruited using a social research company to ensure there was no self-selection bias by individuals interested in nature or well-being and that a diverse public was represented (Supplementary Table 3). All participants were over 18 years old and had been resident in Britain for at least five years. As part of the questionnaire, we asked participants to provide the full postcode of where they lived. We also requested that they indicate a nearby forest on a map and that their questionnaire answers should relate to that forest. We removed data for 513 participants who did not locate a forest from subsequent analyses. We quantified the well-being people associate with forest biodiversity using BIO-WELL, a biodiversity–well-being psychometric scale^18^ (https://research.kent.ac.uk/bio-well/). Participants were asked to record their well-being (physical, emotional, cognitive, spiritual and social) responses to different metrics and attributes of biodiversity (Supplementary Text provides details). For each participant, we calculated mean overall BIO-WELL scores across physical, cognitive, emotional, social and spiritual well-being.

### Spatio-temporal distributions of BIO-WELL scores

We also examined whether there were differences in participants’ BIO-WELL scores between the NFI categories associated with their nearby forests or across the seasons. When determining the NFI category for each participant’s nearby forest, we used a 0.5-km buffer to account for potential resolution errors incurred through the coordinate system used in the online questionnaire. The differences were tested statistically using a Kruskal Wallis H and post-hoc Dunn–Bonferroni tests adjusted for multiple comparisons.

### Effect traits, well-being and socio-economic deprivation

We used a government dataset to assess levels of human socio-economic deprivation^77^, at the smallest possible spatial resolution of Lower Super Output Areas (LSOAs; with an average of 1,700 people per LSOA^78^). Within each LSOA, we used the proportion of the population living in income- or employment-related deprivation (Extended Data Fig. 2). For each season, we then extracted the mean cumulative species’ effect trait richness value of all forests within the LSOA, using the ‘extract’ function in the package Raster^67^. We related the mean cumulative species’ effect trait richness of forests to the two measures of deprivation using a general linear model with a negative binomial error distribution. We included forest area (ha) as a covariate, given that larger areas are expected to contain a higher diversity of species^79^. Before this, we checked to ensure variance inflation factors were below 1.7 (ref. ^80^) and ran a bivariate linear model to investigate whether more deprived areas have smaller forests. This approach was repeated for participants’ BIO-WELL scores and deprivation. All models were checked for fit, overdispersion and homoscedasticity^81^.

### Reporting summary

Further information on research design is available in the Nature Portfolio Reporting Summary linked to this article.

## Supplementary information

Supplementary Information Supplementary Tables 1–8, text and BIO-WELL questions.

Reporting Summary

## Data Availability

The authors confirm that all questionnaire data generated during this study can be accessed at 10.22024/UniKent/01.01.541. All species and environmental data analysed for country-wide modelling during this study are freely accessible as detailed in the Supplementary Material.
