# Supplementary material for: Spatio-temporal variability in forest biodiversity associated with human well-being across socio-economic deprivation gradients
Source: Nat Ecol Evol. 2025 Jun 24;9(8):1382–92. doi: 10.1038/s41559-025-02765-w (PMC12328225; doi:10.1038/s41559-025-02765-w)
Supplement: Supplementary file 1 — Supplementary Tables 1–8, text and BIO-WELL questions. [file 41559_2025_2765_MOESM1_ESM.pdf]

nature ecology & evolution

Supplementary information

https://doi.org/10.1038/s41559-025-02765-w

Spatio-temporal variability in forest biodiversity associated with human well-being across socio-economic deprivation gradients

In the format provided by the authors and unedited

Supplementary Information

Supplementary Table 1 | National Forest Inventory (NFI) definitions of forest categories (www.forestry.gov.uk/inventory).

Our forest category	NFI forest category	NFI definition
Broadleaf	Broadleaved	The canopy of broadleaved forest is generally more uneven than that of coniferous forest being made up of rounded crowns but with variations according to species, age, height, and season. Boundaries with adjacent internal polygons are generally less clearly defined than with conifers and naturally occurring stands may grade into adjacent ones with no sharp division. Some coniferous trees may be present but greater than 80% of the area will consist of broadleaved trees
Coniferous	Conifer	Coniferous forest often occurs as large plantations with trees in regular rows and the stand edges may be regular and sharply defined. Some broadleaved trees may also be present but greater than 80% of the area will consist of conifers.
Mixed (mainly broadleaved)	Mixed predominantly broadleaved	The interpretation of Mixed forest can be very difficult as it exhibits intermediate characteristics between Conifer and Broadleaved forest. There can be several types of mixed forest. A plantation of alternate rows of conifer and broadleaves may produce a 'striped' appearance. You may see conifer and broadleaves planted in blocks, and there may be general intersperse forest. The proportion of the Broadleaves will be more than 50% of the area and less than 80%.
Mixed (mainly conifer)	Mixed predominantly conifer	The interpretation of Mixed forest can be very difficult as it exhibits intermediate characteristics between Conifer and Broadleaved forest. There can be several types of mixed forest. A plantation of alternate rows of conifer and broadleaves may produce a 'striped' appearance. You may see conifer and broadleaves planted in blocks, and there may be general intersperse forest. The proportion of the Conifer will be more than 50% of the area and less than 80%.
Other	Assumed forest	The supplied grant scheme and FC new planting polygons have been attributed as 'assumed forest' as these areas have not been checked against the latest images and will be monitored in future updates and either updated to reflect forest type or removed from the dataset.
	Low density	This category intended to include areas that have less than 20% canopy cover that might have potential to achieve forest in the future. These

	areas will be monitored in future updates and either updated to reflect forest type or removed from the dataset.
Uncertain	Where the interpreter is uncertain of the IFT/IOA to be used X will be designated. The rate of use of this category should decline over time as operators become more proficient and better at recognising IFT/IOAs. As part of the QA procedures X's will be checked and operators found using this code frequently will be subject to more intensive QA procedures and possibly more training.
Cloud or shadow	If cloud or shadow areas obscure forest detail and it is difficult to allocate one of the above IFTs, and then digitise a new boundary line feature around the area of uncertain forest type.
Ground prepared for new planting	Land in this category is area recently converted from some other land use to forest and will show plough furrows or mounding but the new planting (if present) cannot yet be discerned
Felled	Areas of forest where the trees have been harvested or felled. Stumps or felled trees may be visible and there may be long heaps of felling debris ('windrows'). Some standing trees within this limit may also be present but should be disregarded. The areas concerned may also have been re-stocked but the new trees are not yet visible.
Young trees	Areas where planting is clearly visible but the trees cannot yet be allocated between Conifer and Broadleaved due to their immaturity. These areas can be on either land new to forest or where a felled crop has been replaced.
Shrub	This category is intended to include areas that may possibly be forest, where the growth is close to the ground and shows a rough character but no clear differentiation between Conifer and Broadleaved can yet be made. Areas being colonised by woody species may fall into this category. The cover will be at least 20%.
Coppice-with-standards	Some areas of coppice also include larger broadleaved trees set in the coppice matrix. These broadleaved trees, often oak, are known as standards and show very clearly over the even coppice as large rounded crowns. The distribution of the standards may also be fairly scattered with approximately 25 per ha.
Coppice	The most important characteristic of coppice areas on aerial photography is very even, smooth appearance. The coppice area may be made up of a patchwork of different ages (heights) but all show this very even character. Areas recently cut may appear to have a very clear floor with little felling debris.

Supplementary Table 2 | Differences in mean cumulative species' effect trait richness, for positive and negative wellbeing separately, between forest categories. Dunn-Bonferroni tests adjusted for multiple comparisons, with the *z* statistic and levels of significance provided (*, p<0.05; **, p<0.01; ***, p<0.001; highlighted in bold).

**Wellbeing response**	**Season**	**Forest category**	Broadleaved	Conifer	Mixed (mainly broadleaf)	Mixed (mainly conifer)
**Positive**	**Autumn**	Conifer	**92.590*** **			
		Mixed (mainly broadleaf)	**18.248*** **	**-33.391*** **		
		Mixed (mainly conifer)	**4.614*** **	**-46.098*** **	**-10.111*** **	
		Other	**151.872*** **	**36.118*** **	**57.425*** **	**70.647*** **
	**Winter**	Conifer	**137.399*** **			
		Mixed (mainly broadleaf)	**48.721*** **	**-29.552*** **		
		Mixed (mainly conifer)	**30.024*** **	**-46.998*** **	**-13.910*** **	
		Other	**210.514*** **	**42.011*** **	**57.175*** **	**75.314*** **
	**Spring**	Conifer	**58.312*** **			
		Mixed (mainly broadleaf)	**5.856*** **	**-26.238*** **		
		Mixed (mainly conifer)	**-6.942*** **	**-38.129*** **	**-9.471*** **	
		Other	**121.109*** **	**42.602*** **	**54.116*** **	**66.501*** **
	**Summer**	Conifer	**-21.961*** **			
		Mixed (mainly broadleaf)	**-24.891*** **	**-11.082*** **		
		Mixed (mainly conifer)	**-32.147*** **	**-17.751*** **	**-5.318*** **	
		Other	**17.066*** **	**32.833*** **	**32.240*** **	**39.199*** **
**Negative**	**Autumn**	Conifer	**69.869*** **			
		Mixed (mainly broadleaf)	**3.275** **	**-34.895*** **		
		Mixed (mainly conifer)	**-5.342*** **	**-42.937*** **	**-6.376*** **	
		Other	**124.141*** **	**34.692*** **	**34.692*** **	**66.468*** **

**Winter**	Conifer	**126.858*** **			
	Mixed (mainly broadleaf)	**39.713*** **	**-32.154*** **		
	Mixed (mainly conifer)	**23.717*** **	**-47.090*** **	**-11.898*** **	
	Other	**196.701*** **	**40.611*** **	**58.985*** **	**74.523*** **
**Spring**	Conifer	**51.866*** **			
	Mixed (mainly broadleaf)	1.427	**-26.832*** **		
	Mixed (mainly conifer)	**-9.026*** **	**-36.548*** **	**-7.729*** **	
	Other	**111.788*** **	**41.064*** **	**53.759*** **	**63.888*** **
**Summer**	Conifer	**-28.462*** **			
	Mixed (mainly broadleaf)	**-30.739*** **	**-12.958*** **		
	Mixed (mainly conifer)	**-36.099*** **	**-17.865*** **	**-3.905*** **	
	Other	**7.704*** **	**31.312*** **	**33.222*** **	**38.353*** **

Supplementary Table 3 | Demographic and socioeconomic characteristics of participants who completed our online questionnaire (*N*=4197) across England and Wales during each season (autumn, n=1020; winter, n=1115; spring, n=1021; summer, n=1041). Categorical (*n* and %) and continuous (median and range) participant-level data.

**Categorical variables**	**Autumn**		**Winter**		**Spring**		**Summer**	
	n	**%**	***N***	**%**	***n***	**%**	***n***	**%**
Gender (male)	576	56.47	580	52.02	517	50.64	547	52.55
Gender (female)	444	43.53	535	47.98	504	49.36	494	47.45
Ethnicity (white British)	812	79.61	870	78.03	765	74.93	760	73.01
Ethnicity (other)	208	20.39	245	21.97	256	25.07	281	26.99
Education (higher)	426	41.76	476	42.69	452	44.27	473	45.44
Country of residence (England)	514	50.39	600	53.81	610	59.75	570	54.76
Country of residence (Wales)	506	49.61	515	46.19	411	40.25	471	45.24
Nearby forest category (broadleaf)	819	80.29	920	82.51	827	81.00	841	80.79
Nearby forest category (conifer)	79	7.75	79	7.09	91	8.91	78	7.49
Nearby forest category (mixed - mainly broadleaf)	38	3.73	25	2.24	34	3.33	31	2.98
Nearby forest category (mixed - mainly conifer)	19	1.86	11	0.99	12	1.18	17	1.63
Nearby forest category (other)	65	6.37	80	7.17	57	5.58	74	7.11
**Continuous variable**	**Median**	**Range**	**Median**	**Range**	**Median**	**Range**	**Median**	**Range**
Age	43	18 - 88	47	18 - 93	48	18 - 85	47	18 - 84

Supplementary Table 4 | Differences in participant BIO-WELL scores between forest categories. Participants indicated the location of the nearby forest their BIO-WELL score was relevant to in the questionnaire, which was subsequently categorised. Dunn-Bonferroni tests adjusted for multiple comparisons, with the *z* statistic and levels of significance provided (*, p<0.05; **, p<0.01; ***, p<0.001; highlighted in bold).

**Season**	**Forest category**	Broadleaf	Conifer	Mixed (mainly broadleaf)	Mixed (mainly conifer)
**Autumn**	Conifer	-0.724			
	Mixed (mainly broadleaf)	0.547	0.894		
	Mixed (mainly conifer)	0.557	0.844	0.132	
	Other	-0.982	-0.258	-1.070	-0.991
**Winter**	Conifer	-0.455			
	Mixed (mainly broadleaf)	-1.003	-0.646		
	Mixed (mainly conifer)	-0.140	0.025	0.411	
	Other	-1.188	-0.537	0.270	-0.276
**Spring**	Conifer	-1.205			
	Mixed (mainly broadleaf)	-0.189	0.530		
	Mixed (mainly conifer)	0.987	1.363	0.964	
	Other	0.428	1.123	0.433	-0.714
**Summer**	Conifer	**-2.783***			
	Mixed (mainly broadleaf)	-0.687	0.947		
	Mixed (mainly conifer)	0.028	1.148	0.413	
	Other	-0.951	1.284	0.039	-0.426

Supplementary Table 5 | Associations between mean cumulative species' effect trait richness and participant BIO-WELL scores, per season, with socioeconomic deprivation in the areas where participants live in England and Wales. General linear models of (**a**) cumulative species' effect trait richness, for positive and negative wellbeing separately, in the area where participants live, for each season, and income-/employment-related deprivation and (**b**) BIO-WELL scores in response to forest biodiversity, for each season, and income-/employment-related deprivation. Models include forest area (ha) as a covariate. Relationships are considered significant when the 95% confidence interval does not cross zero (highlighted in bold).

**(a) Effect trait richness**	**Autumn**			**Winter**			**Spring**			**Summer**		
**Predictors**	β	2.50%	97.50%	β	2.50%	97.50%	β	2.50%	97.50%	β	2.50%	97.50%
**Positive wellbeing**												
Income-related deprivation	**-1.559**	**-2.024**	**-1.087**	**-1.491**	**-2.005**	**-0.968**	**-1.234**	**-1.772**	**-0.685**	**-1.211**	**-1.659**	**-0.758**
Forest area (ha)	0.000	-0.000	0.000	0.000	-0.000	0.000	0.000	-0.000	0.000	0.000	-0.000	0.000
Employment-related deprivation	**-2.910**	**-3.471**	**-2.340**	**-2.723**	**-3.367**	**-2.067**	**-2.707**	**-3.377**	**-2.020**	**-2.133**	**-2.672**	**-1.586**
Forest area (ha)	0.000	-0.000	0.000	0.000	-0.000	0.000	0.000	-0.000	0.000	0.000	-0.000	0.000
**Negative wellbeing**												
Income-related deprivation	**-1.557**	**-2.040**	**-1.068**	**-1.660**	**-2.248**	**-1.067**	**-1.494**	**-2.157**	**-0.817**	**-1.441**	**-1.962**	**-0.916**
Forest area (ha)	**0.000**	**0.000**	**0.000**	0.000	-0.000	0.000	0.000	-0.000	0.000	0.000	-0.000	0.000
Employment-related deprivation	**-2.838**	**-3.427**	**-2.245**	**-2.807**	**-3.547**	**-2.062**	**-3.312**	**-4.148**	**-2.458**	**-2.541**	**-3.174**	**-1.902**
Forest area (ha)	**0.000**	**-0.000**	**0.000**	0.000	-0.000	0.000	0.000	-0.000	0.000	0.000	-0.000	0.000

(b) BIO-WELL scores	Autumn			Winter			Spring			Summer		
**Predictors**	β	2.50%	97.50%	β	2.50%	97.50%	β	2.50%	97.50%	β	2.50%	97.50%
Income-related deprivation	-0.061	-0.224	0.102	**-0.210**	**-0.369**	**-0.051**	**-0.091**	**-0.262**	**-0.081**	-0.058	-0.237	0.121
Forest area (ha)	0.000	0.000	0.000	0.000	0.000	0.000	0.000	0.000	0.000	0.000	0.000	0.000
Employment-related deprivation	-0.055	-0.262	0.151	-0.185	-0.397	0.027	-0.032	-0.189	0.254	0.013	-0.214	0.240
Forest area (ha)	0.000	0.000	0.000	0.000	-0.000	0.000	0.000	0.000	0.000	0.000	0.000	0.000

Supplementary Table 6 | Associations between mean cumulative species' effect trait richness, per season, and socioeconomic deprivation across all of England and Wales. General linear models of (**a**) mean cumulative species' effect trait richness per LSOA, for each season, and income-/employment-related deprivation, for positive and negative wellbeing separately. Models include forest area (ha) as a covariate. Relationships are considered significant when the 95% confidence interval does not cross zero (highlighted in bold).

**(a) Effect trait richness**	**Autumn**			**Winter**			**Spring**			**Summer**		
**Predictors**	β	2.50%	97.50%	β	2.50%	97.50%	β	2.50%	97.50%	β	2.50%	97.50%
**Positive wellbeing**												
Income-related deprivation	**-2.619**	**-2.658**	**-3.643**	**-2.092**	**-2.136**	**-2.049**	**-3.844**	**-3.883**	**-3.804**	**-3.130**	**-3.165**	**-3.094**
Forest area (ha)	**-0.001**	**-0.001**	**-0.000**	**-0.001**	**-0.001**	**-0.001**	**-0.000**	**-0.000**	**-0.000**	**0.000**	**0.000**	**0.000**
Employment-related deprivation	**-3.731**	**-3.779**	**-3.683**	**-3.043**	**-3.097**	**-2.990**	**-5.416**	**-5.463**	**-5.369**	**-4.127**	**-4.169**	**-4.084**
Forest area (ha)	**-0.001**	**-0.001**	**-0.000**	**-0.001**	**-0.001**	**-0.001**	**-0.000**	**-0.000**	**-0.000**	**0.000**	**0.000**	**0.000**
**Negative wellbeing**												
Income-related deprivation	**-2.653**	**-2.694**	**-2.612**	**-2.590**	**-2.640**	**-2.541**	**-4.562**	**-4.612**	**-4.513**	**-3.589**	**-3.630**	**-3.548**
Forest area (ha)	**-0.000**	**-0.000**	**-0.000**	**-0.001**	**-0.001**	**-0.001**	**-0.000**	**-0.000**	**-0.000**	**-0.000**	**-0.000**	**-0.000**
Employment-related deprivation	**-3.693**	**-3.744**	**-3.643**	**-3.781**	**-3.842**	**-3.720**	**-6.530**	**-6.591**	**-6.470**	**-4.718**	**-4.768**	**-4.667**
Forest area (ha)	**-0.000**	**-0.000**	**-0.000**	**-0.001**	**-0.001**	**-0.001**	**-0.000**	**-0.000**	**-0.000**	**-0.000**	**-0.000**	**-0.000**

Supplementary Table 7 | Data providers of presence/absence records used to generate the species distribution models (SDMs). Common and Latin name for each species (*n*=131) for which SDMs were generated.

Common name	Latin name	Common name	Latin name
**Birds (*n*=58)**			
British Trust for Ornithology (BTO/Joint Nature Conservation Committee/Royal Society for the Protection of Birds partnership). Records provided by BTO, accessed through NBN Atlas website. https://registry.nbnatlas.org/public/showDataResource/dr2370			
black grouse	*Lyrurus tetrix*	magpie	*Pica pica*
Blackcap	*Sylvia atricapilla*	Mandarin duck	*Aix galericulata*
Canada goose	*Branta canadensis*	marsh tit	*Poecile palustris*
Chiffchaff	*Phylloscopus collybita*	nightingale	*Luscinia megarhynchos*
collared dove	*Streptopelia decaocto*	nuthatch	*Sitta europaea*
Crossbill	*Loxia curvirostra*	pied flycatcher	*Ficedula hypoleuca*
Dunnock	*Prunella modularis*	pied wagtail	*Motacilla alba*
Fieldfare	*Turdus pilaris*	red kite	*Milvus milvus*
Firecrest	*Regulus ignicapilla*	redstart	*Phoenicurus phoenicurus*
garden warbler	*Sylvia borin*	redwing	*Turdus iliacus*
Goldcrest	*Regulus regulus*	ring-necked parakeet	*Psittacula krameri*
Goldfinch	*Carduelis carduelis*	rock dove	*Columba livia*
Goshawk	*Accipiter gentilis*	siskin	*Spinus spinus*
great spotted woodpecker	*Dendrocopos major*	song thrush	*Turdus philomelos*
great tit	*Parus major*	sparrowhawk	*Accipiter nisus*
green woodpecker	*Picus viridis*	spotted flycatcher	*Muscicapa striata*
Greenfinch	*Chloris chloris*	starling	*Sturnus vulgaris*
grey heron	*Ardea cinerea*	stock dove	*Columba oenas*
Hawfinch	*Coccothraustes coccothraustes*	tree pipit	*Anthus trivialis*
Hobby	*Falco subbuteo*	tree sparrow	*Passer montanus*
hooded crow	*Corvus cornix*	turtle dove	*Streptopelia turtur*
Jay	*Garrulus glandarius*	waxwing	*Bombycilla garrulus*
Kestrel	*Falco tinnunculus*	willow tit	*Poecile montanus*
lesser redpoll	*Acanthis cabaret*	wood warbler	*Phylloscopus sibilatrix*
lesser spotted woodpecker	*Dryobates minor*	woodcock	*Scolopax rusticola*
lesser whitethroat	*Curruca curruca*	woodlark	*Lullula arborea*
little owl	*Athene noctua*	woodpigeon	*Columba palumbus*

long-eared owl	*Asio otus*	wren	*Troglodytes troglodytes*
long-tailed tit	*Aegithalos caudatus*	yellow-browed warbler	*Phylloscopus inornatus*

Butterflies (*n*=21)

Records provided by the UK Butterfly Monitoring Scheme (UKBMS), a partnership between Butterfly Conservation, Centre for Ecology & Hydrology, British Trust for Ornithology, and the Joint Nature Conservation Committee, accessed through NBN Atlas.
https://registry.nbnatlas.org/public/showDataResource/dr1206

Brimstone	*Gonepteryx rhamni*	orange-tip	*Anthocharis cardamines*
brown argus	*Aricia agestis*	painted lady	*Vanessa cardui*
Comma	*Polygonia c-album*	peacock	*Aglais io*
common blue	*Polyommatus icarus*	red admiral	*Vanessa atalanta*
dark green fritillary	*Speyeria aglaja*	silver-washed fritillary	*Argynnis paphia*
Essex skipper	*Thymelicus lineola*	small copper	*Lycaena phlaeas*
Gatekeeper	*Pyronia tithonus*	small skipper	*Thymelicus sylvestris*
holly blue	*Celastrina argiolus*	small tortoiseshell	*Aglais urticae*
large skipper	*Ochlodes sylvanus*	small white	*Pieris rapae*
large white	*Pieris brassicae*	white admiral	*Limenitis camilla*
marbled white	*Melanargia galathea*		

Fungi (*n*=6)

Records provided by the Fungal Records Database of Britain and Ireland (FRDBI). Reference: The British Mycological Society, (2009). Fungal Records Database of Britain and Ireland. Cannon, P. (1998). Database of British fungal records from literature sources. Mycologist 12:25-26.

amethyst deceiver	*Laccaria amethystina*	lilac bonnet	*Mycena pura*
angel's bonnet	*Mycena arcangeliana*	stump puffball	*Apioperdon pyriforme*
blushing bracket	*Daedaleopsis confragosa*	sulphur tuft	*Hypholoma fasciculare*

Mammals (*n*=3)

Records provided by The Mammal Society, accessed through NBN Atlas.
https://registry.nbnatlas.org/public/show/dp8

roe deer	*Capreolus capreolus*	West European hedgehog	*Erinaceus europaeus*
European fox	*Vulpes vulpes*		

Plants (trees and shrubs) (*n*=43)

Records provided by the Botanical Society of Britain and Ireland (BSBI). Reference: Pescott, O. L., Humphrey, T. A., & Walker, K. J. (2018). A Short Guide to Using British and Irish Plant Occurrence Data for Research. nora.nerc.ac.uk..

Aspen	*Populus tremula*	horse chestnut	*Aesculus hippocastanum*

bird cherry	*Prunus padus*	Lawson cypress	*Chamaecyparis lawsoniana*
black pine	*Pinus nigra*	lodgepole pine	*Pinus contorta*
Blackthorn	*Prunus spinosa*	Norway maple	*Acer platanoides*
cherry plum	*Prunus cerasifera*	Norway spruce	*Picea abies*
common beech	*Fagus sylvatica*	osier willow	*Salix viminalis*
common box	*Buxus sempervirens*	rowan	*Sorbus aucuparia*
common hawthorn	*Crataegus monogyna*	Scots pine	*Pinus sylvestris*
common holly	*Ilex aquifolium*	sessile oak	*Quercus petraea*
copper beech	*Fagus sylvatica f. purpurea*	silver birch	*Betula pendula*
Dogwood	*Cornus sanguinea*	Sitka spruce	*Picea sitchensis*
downy birch	*Betula pubescens*	small-leaved lime	*Tilia cordata*
English elm	*Ulmus procera*	spindle	*Euonymus europaeus*
English oak	*Quercus robur*	sweet chestnut	*Castanea sativa*
European larch	*Larix decidua*	sycamore	*Acer pseudoplatanus*
field maple	*Acer campestre*	turkey oak	*Quercus cerris*
goat willow	*Salix caprea*	white willow	*Salix alba*
grey willow	*Salix cinerea subsp. oleifolia*	whitebeam	*Sorbus aria*
guelder rose	*Viburnum opulus*	wild cherry	*Prunus avium*
Hazel	*Corylus avellana*	wych elm	*Ulmus glabra*
holm oak	*Quercus ilex*	yew	*Taxus baccata*
Hornbeam	*Carpinus betulus*		

Supplementary Table 8 | Predictor variables used in the species distribution models (SDMs). Variables were selected that were biologically meaningful to the presence of forest species, and sampled at 0.5 km resolution (30 arc-seconds).

**Variable**	**Description**	**Reference**
Topsoil – land cover	Derived from the Land Cover Map (2007). Dominant land cover at broad habitat level (e.g. coniferous forest)	Emmett, B.A., Reynolds, B., Chamberlain, P.M., Rowe, E., Spurgeon, D., Brittain, S.A., Frogbrook, Z., Hughes, S., Lawlor, A.J. and Poskitt, J., (2010). Countryside Survey: Soils Report from 2007: CS Technical Report No. 9/07. Centre for Ecology & Hydrology: Wallingford, UK.
Topsoil – dominant grain size	A classification of the most common (dominant) grain size (mm) to be expected from the parent material	
Topsoil – mean soil nitrogen concentration	Mean value for total soil nitrogen concentration in 2007 modelled by LCM_CLASS and CACO3_RANK (Classification of all forms of carbonate content in each parent material (calcite, dolomite, siderite) as a ranking of: none, low, medium or high (with unknown or variable for heterolithic and multilithic parent units)	
BioClim	Annual mean temperature, Maximum temperature of the warmest month, Minimum temperature of the coldest month, Temperature annual range, Annual precipitation, Precipitation seasonality (coefficient of variation), Precipitation of the driest quarter, Precipitation of the warmest quarter, elevation	Hijmans, R. J., Cameron, S. E., Parra, J. L., Jones, P. G., & Jarvis, A. (2005). Very high resolution interpolated climate surfaces for global land areas. International Journal of Climatology, 25, 1965–1978

Supplementary Text: BIO-WELL questions

For further information, go to https://research.kent.ac.uk/bio-well/ and see Irvine, K.N., Fisher, J.C., Bentley, P.R., Nawrath, M., Dallimer, M., Austen, G.E., Fish, R. and Davies, Z.G., (2023). BIO-WELL: the development and validation of a human wellbeing scale that measures responses to biodiversity. Journal of Environmental Psychology, 85.

For each of the following questions, imagine yourself in the nearby forest at this time of year. Please think about the living things, including the plants, fungi and animals (but not pets, horses, cows, sheep), in that forest.

BIO-WELL comprises 17 biodiversity stem questions, each one capturing a metric/attribute of biodiversity:

	**Metric/Attribute**
**Biodiversity metric stem questions**	
1. Encountering the living things (e.g. plants, fungi and animals) in this forest makes me feel…	Encountering
2. The number of living things (e.g. plants, fungi and animals) in this forest makes me feel…	Abundance
3. The variety of living things (e.g. plants, fungi and animals) in this forest makes me feel…	Species diversity
4. The interactions between plants, fungi and animals (e.g. pollination, predator-prey) in this forest make me feel…	Species interactions
5. The living processes (e.g. decomposing, growing) that happen in this forest make me feel…	Biological processes
**Biodiversity attribute stem questions**	
6. The variety of sounds in this forest make me feel…	Sound
7. The distinctive sounds in this forest make me feel…	
8. The variety of colours in this forest make me feel…	Colour
9. The vivid colours in this forest make me feel…	
10. The variety of shapes in this forest make me feel…	Shape
11. The maturity of living things (e.g. plants, fungi and animals) in this forest makes me feel…	
12. The variety of textures in this forest make me feel…	Texture
13. The sponginess of living things (e.g. plants, fungi and animals) in this forest makes me feel…	
14. The variety of smells in this forest make me feel…	Smell
15. The woody smells in this forest make me feel…	
16. Changes in this season make me feel…	Behaviour
17. The presence of animals in this forest makes me feel…	

Participants must complete five wellbeing response items, using a visual analogue scale from 0 to 100, for each biodiversity stem question:

1. Physically relaxed (0) – Physically tense (100) [representing the physical wellbeing domain]
2. Joyful (0) – Sad (100) [emotional wellbeing]
3. Clear minded (0) – Muddled (100) [cognitive wellbeing]
4. Open to people (0) – Closed to people (100) [social wellbeing]
5. Part of something bigger than myself (0) – Not part of something bigger than myself (100) [spiritual wellbeing]
